# Integrated Assessment of Potentially Toxic Elements (PTEs) Pollution in Agricultural Soils of North Gondar Zone, Ethiopia: Physicochemical Parameters, Pollution Levels, and Associated Health Risks

**DOI:** 10.3390/toxics14070613

**Published:** 2026-07-13

**Authors:** Teferi Aschalew Nega, Mihret Kendie Wolie, Enkuahone Abiyu Kassa, Alemken Berie Teshager, Kenaw Abeye Adimasu, Weiying Feng, Chia Min Ho

**Affiliations:** 1 School of Material Science and Engineering, Beihang University, Beijing 100191, China; 2 Department of Chemistry, Debark University, Debark 90, Ethiopia

**Keywords:** agricultural soils, carcinogenic risk, potentially toxic elements, physicochemical parameters, pollution indices, sustainable soil management

## Abstract

Agricultural soil contamination by potentially toxic elements is a global concern due to its impacts on food safety and human health, yet comprehensive assessments remain limited in many regions of Ethiopia. This study provides an integrated assessment of PTE contamination in agricultural soils of the North Gondar Zone, Ethiopia, by evaluating physicochemical properties, pollution levels, and human health risks. Soil parameters, including pH, electrical conductivity, organic carbon, organic matter, moisture content, total nitrogen, and available phosphorus, varied among sampling sites. Soil pH ranged from moderately acidic to near-neutral, indicating variations in soil acidity likely associated with differences in moisture content, organic matter, and land management practices, while electrical conductivity values indicated non-saline conditions suitable for agriculture. Concentrations of PTEs (As, Zn, Cd, Pb, and Hg) were generally within permissible limits established by WHO and FAO. Pollution indices revealed predominantly natural background levels for As, Zn, Cd, and Pb, whereas Hg exhibited moderate to strong contamination, with the Geoaccumulation Index and Contamination Factor identifying Hg as the primary environmental risk element. Non-carcinogenic risk assessment showed that hazard quotients and hazard indices for both adults and children were below 1, indicating negligible health risks. Carcinogenic risk assessment demonstrated that all calculated risks were within the acceptable range (10^−6^–10^−4^), although children showed higher total cancer risk (TCR) values than adults due to greater exposure intensity and lower body weight. Arsenic was identified as the dominant contributor to carcinogenic risk across all sampling sites. The findings demonstrate that agricultural soils in the study area are generally safe with respect to the investigated PTEs; however, Hg contamination indices indicate a potential environmental concern requiring continued monitoring. Sustainable soil management practices, including effective pH management and liming of strongly acidic soils (pH < 5.5), are recommended to improve soil quality, reduce PTE mobility and bioavailability, and minimize future accumulation of hazardous elements while maintaining agricultural productivity.

## 1. Introduction

Soil is as a complex, living, continuously shifting and dynamic natural system composed of minerals, organic matter, water, and air, forming the uppermost layer of the earth, and it functions as a fundamental natural resource for terrestrial ecosystems [1]. It supports plant growth, regulates the movement of nutrients and minerals through biogeochemical cycles, and plays a critical role in global food production systems [2,3]. Agricultural soils are fundamental to food production, ecosystem sustainability, and human well-being. However, increasing natural and anthropogenic activities have led to the accumulation of PTEs in soil, which can reduce soil fertility, contaminate food crops, and pose risks to environmental and public health [4]. PTEs are among the most significant soil contaminants worldwide because they persist in the environment, do not biodegrade, and can bioaccumulate in living organisms through the food chain [5,6]. Elements such as Cd, Pb, Ni, Cu, Fe, As, Hg, and Zn are naturally present in soil but may reach toxic levels through anthropogenic activities. These PTEs are characterized by relatively high atomic density (>5 g/cm^3^) and can exhibit toxicity even at low concentration levels [7]. Their accumulation degrades soil quality, disrupts microbial and ecological processes, reduces crop safety, and poses significant health risks to both humans and animals [8].

PTEs in soil originate from both natural and anthropogenic sources. Natural sources include lithogenesis, weathering of parent rock, erosion, sediment transport, and volcanic activity, whereas anthropogenic sources are linked with transportation, fertilizers, pesticides, mining, industrial activities and improper waste disposal [9,10,11]. Human exposure to PTEs can result from various factors, including dermal contact, ingestion of contaminated food, and exposure to particular matter [12]. The deposition of PTEs in soil is a significant concern because these PTEs can be absorbed by plants, enter the food chain, and pose risks to both the environment and human health [13,14]. The elevated levels of PTEs in both soil and aquatic environments are a significant cause for concern, as they act as environmental toxins [15,16]. Although several PTEs serve as micronutrients al low concentrations, excessive accumulation can significantly change soil physicochemical properties and reduce soil functional stability [17]. PTEs such as Cd, Pb, Hg, As, Cr, and Co inhibit plant growth, impair microbial activity, and disturb soil ecological balance even at low exposure levels. The environmental fate and transport of PTES in soils are strongly influenced by physicochemical properties such as pH, organic matter content, electrical conductivity, and soil texture [18]. In humans, chronic exposure is associated with neurological, renal, cardiovascular, respiratory, reproductive, and carcinogenic effects [19]. Fertilizers can act as an important anthropogenic source of PTEs in agricultural soils. Phosphate fertilizers are primarily associated with cadmium (Cd) enrichment and may also contain trace elements such as Pb, As, and Zn, depending on the geological origin of phosphate rock and manufacturing processes. In addition, organic fertilizers may also contribute multiple metals depending on their origin and treatment conditions [20,21].

In Ethiopia, agriculture is the backbone of the economy, supporting over 80% of the population and contributing approximately 40% of GDP [22]. The sustainability of this sector depends heavily on soil health; however, soil degradation through nutrient depletion, acidification, and contamination poses a significant threat to agricultural productivity and environmental stability [23]. Recent studies have reported elevated PTEs concentrations in soils, water, and crops, particularly in areas influenced by peri-urban and intensively cultivated areas influenced by anthropogenic activities. Agricultural lands in the Amhara region, including areas around Gondar, are increasingly affected by municipal waste, industrial effluents, and agrochemical inputs [24]. The North Gondar Zone, located in the Amhara Region, is characterized by intensive agricultural activities that sustain local livelihoods and contribute significantly to food security in Ethiopia. However, increasing agricultural intensification, population growth, and increasing use of agrochemicals and irrigation sometimes from untreated sources have raised concerns about PTEs accumulation in soils. Although the area plays a vital role in food security and livelihoods, comprehensive assessments of soil contamination remain limited.

There is a critical need for a comprehensive and integrated assessment systematically combines PTEs quantification, soil physicochemical analysis, pollution indexing, and human health risk evaluation within a unified analytical framework. Using this approach would make contamination assessments more reliable and more useful for guiding environmental management and policy decisions. However, integrated studies addressing PTEs contamination, soil physicochemical properties, and associated human health and ecological risks are not studied in agricultural soils of the North Gondar Zone. Therefore, this study aims to provide an integrated assessment of PTES pollution in the agricultural soils of the North Gondar Zone. The objectives of the study are: (i) to analyze key soil physicochemical properties; (ii) to determine the concentrations of selected PTES; (iii) To assess Pollution assessment indices the levels of PTEs in agricultural soil samples and (iv) To evaluate the non-carcinogenic and carcinogenic human health risk associated with the PTEs for adults and children. The findings of this study will provide valuable baseline data for policymakers, environmental managers, and researchers, supporting the development of effective pollution control measures, sustainable agricultural practices, and public health protection strategies.

## 2. Materials and Methods

### 2.1. Study Area

**Figure 1:**
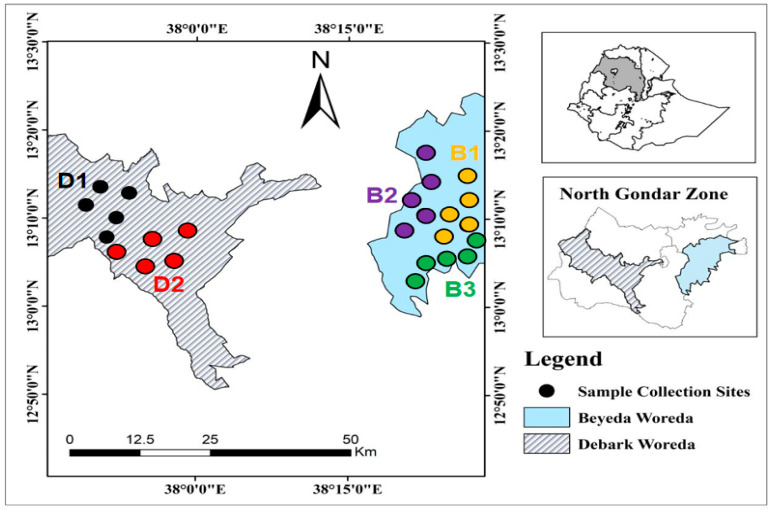
Map of sampling sites.

This research was carried out in two districts of the North Gondar Administrative Zone, namely Beyeda and Debark (Figure 1). Beyeda is located in the eastern part of the North Gondar Zone and is bordered by the Wag Hemra Zone to the south, Jan Amora to the west, Tselemt to the north, and the Tekeze River to the east, which separates it from the Tigray Region. Debark is bordered by Dabat to the south, Tegeda to the west, the Tigray Region to the northwest, Addi Arkay to the north, and Jan Amora to the east. According to the World Reference Base for Soil Resources [25], the dominant soil types in the study area differ between the two districts. In Beyeda Woreda, the major soil types are Eutric Leptosols, Umbric Andosols, Umbric Leptosols, and Eutric Cambisols, whereas in Debark Woreda the dominant soil types are Acrisols, Cambisols, Phaeozems, Lithosols, and Nitisols [26,27]. Five agricultural sampling sites were selected to represent the major cultivated areas of the Debark and Beyeda districts based on differences in geographic location, topography, and agricultural management. The study area is dominated by rain-fed mixed crop–livestock farming, with barley, wheat, faba bean, field pea, and potato as the principal crops. Although the sites share similar climatic conditions and volcanic parent materials, they differ in elevation, slope, and fertilizer and manure management, factors that may influence the spatial distribution of soil physicochemical properties and potentially toxic elements (PTEs). Natural weathering and atmospheric deposition may also contribute to the occurrence of PTEs in the soils.

### 2.2. Sample Collection and Preparation

Soil samples were randomly collected from five sites across two districts of the North Gondar Zone, Ethiopia, between January and March 2024 (G.C.). A total of 25 topsoil samples were collected from five agricultural areas in the Debark and Beyeda districts of the North Gondar Administrative Zone, Ethiopia. Samples were taken from the surface soil layer (0–20 cm depth), which was selected due to its strong influence from agricultural activities such as fertilizer application, irrigation, and pesticide use. After collection, all soil samples were air-dried, gently crushed, and passed through a 2 mm mesh nylon sieve to remove undesired particles (such as stones, residual roots, etc.) and then kept in clean polythene bags. All soil samples were kept dry for PTEs and physiochemical parameter analysis.

### 2.3. Analysis of Physicochemical Parameter in Soil Samples

Physicochemical characterization was carried out on air-dried soil samples in the laboratory. pH was measured in a 1:2.5 (*w*/*v*) soil: water suspension using a calibrated portable digital pH meter (Hach, HQ1110, Loveland, CO, USA) [28]. Electrical conductivity (EC) was determined by stirring soil samples in distilled water at a soil:water ratio of 1/5 *w*/*v* for 30 min with a conductivity meter (Hach, Loveland, CO, USA), following Association of Official Analytical Chemists (AOAC) methods [29]. The moisture content was measured using AOAC methods [29]. The formula below was used to calculate the moisture percentage for each soil sample.

(1) $$Moisture\;content\;(\%)=\frac{W_{s}-\left(Ww_{1}-w_{2}\right)}{W}\times 100$$

where W_S_ is weight of soil sample before heating in a drying oven, W_2_ is final weight of samples and soil sample after drying and W_1_ is initial weight of empty crucible.

Organic matter (OC) content of the soil sample was determined by the procedure reported [30]. The amount of organic carbon (%) and total organic matter (%) were determined by Equations (2) and (3).

(2) $$OC(\%)=\;\frac{N\left(B-S\right)\times 0.003\times f\times 100}{W}$$

OM (%) = OC (%) × 1.724(3)

where N = 0.5 N normality of ferrous ammonium sulfate, B = volume of ferrous ammonium sulfate required for blank (mL), S = volume of ferrous ammonium sulfate required for soil sample (mL) and W = mass of soil sample (g), f = Correction factor (1.33).

Total nitrogen content was determined using the Kjeldahl digestion, distillation, and titration method in accordance with AOAC Official Method [29]. The %N was then calculated as:

(4) $$\;N\left(\%\right)=\frac{Volume\;of\;acid\;(mL)\times Molarity\;of\;acid\times 14\;g/mol}{weight\;of\;sample\;(g)\times \;10}$$

Available phosphorous: The soil’s available phosphorous content was determined by measuring absorbance on a spectrophotometer (UV-1900i, Shimadzu Corporation, Kyoto, Japan) at 400 to 490 nm following the method of extraction according to Bray II [31].

### 2.4. Potentially Toxic Elements (PTES) Metal Analysis in Soils

#### 2.4.1. Sample Digestion and Analysis of PTEs

One gram (1.0 g) of dried, homogenized soil was accurately weighed into a 100 mL round-bottom digestion flask and digested with 15 mL of aqua regia (HCl:HNO_3_, 3:1). The mixture was gently swirled and fitted with a reflux condenser, then heated on a Kjeldahl block (Model KDN-20C, Hinotek, Ningbo, China) at 170–245 °C (optimum 245 °C) for 3 h 30 min until a clear solution was obtained. After digestion, the solution was allowed to cool (10 min with the condenser and 10 min without). After cooling, deionized water was added to dissolve any precipitates, and the digest was filtered through (Whatman®, No. 41, Maidstone, England, UK)paper into a 50 mL volumetric flask and diluted to volume with deionized water. Reagent blanks were prepared following the same procedure. All digests were stored at 4 °C until analysis of PTEs by ICP–OES (PerkinElmer Optima 8000, New York, NY, USA). Each sample was digested in triplicate. Calibration standards were prepared by serial dilution of a 1000 mg/L stock standard solution in 100 mL volumetric flasks, and working standards for individual metals were prepared from the corresponding metal nitrate salts.

#### 2.4.2. Analytical Quality Control

Calibration curves for all elements were established using five different concentration levels. Elemental concentrations in soil samples were quantified using the corresponding linear regression equations derived from the absorbance values. All calibration curves exhibited excellent linearity, with correlation coefficients (R^2^) greater than 0.995, confirming strong analytical performance. Limits of detection (LOD = 3 × SDblank ) and limits of quantification (LOQ = 10 × SDblank) were calculated based on the standard deviation of three replicate blank measurements processed under identical digestion conditions as the samples. The detection limits were sufficiently low to enable the determination of trace-level elements in all soil samples (Table S1). The method demonstrated high accuracy, with percent recoveries for the analyzed PTEs ranging from 96.77% to 108%, which falls within the accepted range of 80–120% for metal analysis (Table S2). Excellent precision was also confirmed, with relative standard deviation (%RSD) values for replicate measurements ranging from 1.49% to 7.07%; all values were below 20%, indicating the reliability and applicability of the analytical method. The recovery percentage was calculated as follows:

(5) $$\mathrm{Recovery}\;(\%)\;=\;\frac{\;C_{spiked\;sample}\;\;-\;\;\;\;C_{unspiked\;sample}}{Actual\;spike\;Concentration\;}\times 100$$

Method precision was assessed as the repeatability of measurements on a homogeneous sample, expressed as the relative standard deviation (RSD).

(6) $$\mathrm{RSD}\;(\%)\;=\frac{\mathrm{Standard}\;\mathrm{deviation}}{\mathrm{Mean}\;\mathrm{value}}\times 100$$

### 2.5. Potentially Toxic Elements (PTEs) Contamination and Pollution Indices Analysis

#### 2.5.1. Methods of Geo-Accumulation Index

The Igeo indexes allow a logarithm operation of the data set for the evaluation of contamination by correlating the obtained current concentration of metals with their background values [32]. The Igeo index value for the metals was determined using the following Equation (7) [33].

(7) $$I_{geo}={log}_{2}\left(\frac{C_{i}}{{1.5B}_{n}}\right)$$

where Ci is the concentration of the measured element, while Bn is the geochemical background value. Factor (1.5) is the background matrix correction factor due to the lithological variations [34]. Igeo ≤ 0: practically free from contamination; 0 ≤ Igeo ≤ 1: free from contamination to moderately polluted; 1 ≤ Igeo ≤ 2: moderately polluted; 2 ≤ Igeo ≤ 3: moderately to severely polluted; 3 ≤ Igeo ≤ 4: severely polluted; 4 ≤ Igeo ≤ 5: severely to extremely polluted; and 5 < Igeo: extremely polluted [33,35]. Background concentrations for Ethiopian highland soils were derived from studies conducted in Amhara, Addis Ababa, and Tigray regions, which have similar volcanic origins and climatic conditions. The combined datasets provided background values of 100 mg/kg for Zn, 0.18 mg/kg for Cd, and 20 mg/kg for Pb, respectively [36,37]. However, due to the lack of localized soil quality guidelines for As and Hg, global average soil concentrations were used, with values of 5 mg/kg for As and 0.05 mg/kg for Hg [38,39].

#### 2.5.2. Methods of Contamination Factor

Contamination factor (CF) was used to evaluate the enrichment of the individual HMs in the soils. The CF is measured from the ration between the obtained concentration of HMs in soil and the background value (concentration in unpolluted soil) for the same metals [40,41].

(8) $$CF=\;\frac{C_{PTEs\;concentration}}{B_{background\;concentration}}$$

#### 2.5.3. Methods of Pollution Load Index

Pollution load index (PLI) is useful to evaluate the level PTEs contamination in Soil [42].

(9) $$PLI=\left({CF}_{1}\times {CF}_{2}\times {CF}_{3}\times \ldots {CF}_{n}\right)\frac{1}{n}$$

where CF is the contamination factor and *n* is the number of HMs studied. The PLI value is less than one (PLI < 1), indicating no metal pollution, PLI = 1, indicating PTEs loads close to the background level [43].

### 2.6. Health Risk Assessments

This study employed the noncancer and cancer health risk assessment model established by USEPA. PTEs in soil samples can be exposed to humans through direct ingestion of soil particles, inhalation of soil particles from the air, and dermal contact with soil particles [44,45]. The estimated chronic daily intake (CDI, mg/kg/day) of PTEs soil was calculated by using the following equations [46,47].

(10) $$CDI(ing)=Cs\times \frac{IngR\times EF\times ED}{BWXAT}\times 10^{-6}$$

(11) $$CDI\left(dermal\right)=Cs\times \frac{SA\times AF\times ABS\times EF\times ED}{BWXAT}\times 10^{-6}$$

where CD (ing) and CDI (dermal) represent the chronic daily intake through ingestion and dermal (mg kg^−1^ day^−1^), respectively; Cs is the concentration of PTEs in soil (mg kg^−1^); IngR is the ingestion rate; EF is the exposure frequency; ED is the exposure duration; SA is the exposed skin surface area; AF is the soil adherence factor; ABS is the dermal absorption fraction; BW is the body weight; AT is the averaging time. The values of all parameters used in the calculations are presented in Table S3.

#### 2.6.1. Noncancer Risk Assessment

The noncancer risk due to PTEs was assessed using the hazard quotient (HQ) and hazard index (HI) parameters. The HQ is calculated as the ratio of the chronic daily intake (CDI, mg/kg/day) to the reference dose (RfD, mg/kg/day). HQ ≥ 1 indicates potential adverse health effects. The reference doses of mg/kg/day values of potentially toxic elements (PTEs) used in the calculations are presented in Table S4.

(12) $$HQi,(ing)=\frac{{CDI}_{i,ing}}{RfD}$$

(13) $$HQi(dermal)=\frac{{CDI}_{i,derm}}{RfD}$$

HQi = Hqi,(ing) + Hqi,(derm)
(14)

HI = ∑HQ = HQ(ing) + HQ(derm)
(15)

#### 2.6.2. Carcinogenic Risk Assessment

The Carcinogenic risk of PTEs was assessed by single-elements Carcinogenic risk (CR) and total Carcinogenic risk (TCR) and calculated by using the following equations:

Cri = CDI × CSF(16)

TCR = ∑CR = CRPb + CRCd + CRAs(17)

where CSF is the cancer slope factor and considered as the risk per (mg/kg/day). The CSF values of Cd, Pb and As are 0.38, 0.0085 and 1.5 in mg/kg/day, respectively.

### 2.7. Statistical Analysis

One-way analysis of variance (ANOVA), followed by Tukey’s post hoc test, was performed to determine significant differences in the mean values of physicochemical properties and potentially toxic element (PTE) concentrations among the sampling sites. Pearson’s correlation analysis was used to evaluate the relationships between the measured variables. Descriptive statistics (mean ± standard deviation) and pollution indices, including the geoaccumulation index (Igeo), contamination factor (CF), and pollution load index (PLI), were calculated. Statistical analyses were conducted using SPSS version 26, while OriginPro 2024b (OriginLab Corporation, Northampton, MA, USA) was used to construct calibration curves and prepare the figures. Statistical significance was established at a 95% confidence level (*p* < 0.05).

## 3. Result and Discussion

### 3.1. Physicochemical Parameter in Soils

**Figure 2:**
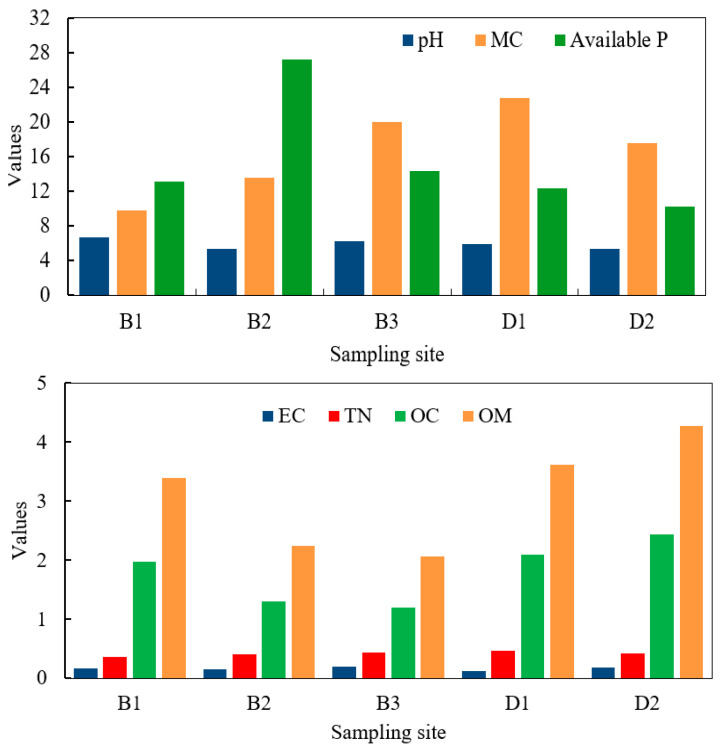
Comparison of physicochemical properties of soil samples from five study areas.

Physicochemical parameters such as pH, EC, OC, OM, MC, TN, and available Phosphorus (AP) were recorded at five sampling sites, as shown in (Table 1 and Figure 2).

The pH values ranged from 5.36 to 6.72. The one-way ANOVA for pH revealed a statistically significant difference among the five sites (*p* < 0.05). However, Tukey’s post hoc test identified statistically significant differences for the following site pairs: B1-B2, B2-B3, B1-D1, B2-D2, B1-D2, and B3-D1 (*p* < 0.05). These moderately acidic to near-neutral pH conditions generally favor the availability of essential nutrients by maintaining balanced soil chemical conditions and supporting moderate rates of mineral weathering. The observed variation in soil pH may be attributed to differences in land use, vegetation cover, organic matter content, and parent material across the study sites. The pH values obtained in this study are within the range of 4.85–6.47 reported for agricultural soils in southwest region of Ethiopia [15]. The pH values measured in this study are lower than those previously reported for soils in Bangladesh (7.53 to 9.24) [48], Nigeria (7.10 to 7.82) [49], and the Wadi al Shati districts of Libya (6.88–7.32) [50].The differences in soil acidity between the studied area and other agricultural regions can be attributed to variations in parent material, rainfall intensity, leaching of basic cations, organic matter decomposition, and land management practices. In the Ethiopian highlands, particularly North Gondar, high rainfall and intense weathering enhance the leaching of base cations (Ca^2+^, Mg^2+^, K^+^), resulting in relatively more acidic soils. Continuous cultivation and limited application of soil amendments such as lime also contribute to increased soil acidity in the study area.

Electrical conductivity (EC) ranged from 0.12 to 0.202 µS/cm. One -way ANOVA showed that there is no significant difference in EC among the five sampling sites (*p* > 0.05). Tukey post hoc tests indicated that there was no significant difference in EC among the pairs B1 to B2, B2 to B3, B1 to D1, B2 to D2, B1 to D2, B3 to D1, and D1 to D2 (*p* > 0.05). These results indicate no statistically significant contrast in EC between these pairwise comparisons. All soils were classified as non-saline, which is typical of rain-fed highland areas with sufficient leaching. The EC values measured here align with those reported for the Wadi al Shati district of Libya (0.14–0.26 μS/cm) [50]. The EC values observed in this study are lower than the reported ranges (103–231 µS/cm) for agricultural soils in the southwest region of Ethiopia [15] and in Peru (225.0–250.3 μS/cm) [51]. The low electrical conductivity of the soil is attributed to the leaching and downward transport of dissolved salts from the soil profile through rainfall and irrigation percolation processes. Soil organic carbon (OC) content ranged from 1.20% to 2.44%. A one-way ANOVA for Organic Carbon indicated a statistically significant difference among the five sites (*p* < 0.05). Tukey post hoc tests identified significant differences in Organic Carbon between the following site pairs: B3-D3, B2-D2, and B3-D1 (*p* < 0.05). No significant differences were observed between B2-B1, B3-B1, B3-B2, D1-B1 and D2-D1 (*p* > 0.05). The organic carbon content observed in this study is higher than the levels reported for soils in the Dhubri district of Assam, India (0.06% to 0.08%) [52]. However, the organic carbon content observed in this study is lower than the reported ranges for soils in Awing, North West Cameroon (2.57–6.78%) [53]. Soil organic matter (OM) content across the study sites ranged from 2.06% to 4.28%. A one-way ANOVA showed significant differences in OM among the five sites (*p* < 0.05). Tukey post hoc tests revealed significant differences in OM between D2-B3, D2-B2, and D1-B3 (*p* < 0.05). In contrast, no significant differences were detected among Paris, B2-B1, B3-B1, D1-B2, D1-B1, and D2-D1 (*p* > 0.05). The OM range measured in this study is comparable to that reported for soils in Nigeria (3.42–4.70%) [49]. The observed values in this study are higher than those reported for the Dhubri district in Assam, India (0.10–0.14%) [52] and for agricultural soils in the southwestern region of Ethiopia (0.68–1.81%) [15]. These differences may reflect variations in crop residue return, manure application, vegetation cover, and decomposition rates, indicating a moderate level of organic matter that is important for soil health, fertility, soil structure, and overall ecosystem function.

The soil moisture content across the study sites ranged from 9.8% to 22.8%. A one-way ANOVA revealed a statistically significant difference in moisture content among the five sites (*p* < 0.05). Tukey’s HSD test showed that site D1 is significantly different from site B1 (*p* < 0.05). However, no other pairwise differences between sites were significant. The moisture content (MC) measured in this study aligns with values reported for southwestern Ethiopia (8.67–20.7%) [15]. In contrast, our values are higher than those reported for Nigeria (2.10–5.23%) [49]. The soil’s moisture content directly affects water availability for plant growth, where both excess and insufficient moisture can negatively influence development and productivity [54,55]. Variations in moisture content in drainage, topography, water retention capacity, and local climatic conditions may contribute to differences in soil properties and nutrient dynamics. The Total Nitrogen value ranged from 0.365% to 0.462%. A one-way ANOVA revealed a statistically significant difference in Total Nitrogen content among the five sites (*p* < 0.05). Post hoc analysis using Tukey’s HSD test indicated that sites B3-B1, D1-B1, and D1-B2 showed statistically significant differences (*p* < 0.05). However, no other pairwise differences between sites were significant. The total nitrogen range measured in this study is comparable to values reported for soils in Poland (0.11–1.45%) [56]. However, the Total Nitrogen content observed in this study is lower than the ranges reported in Punjab, India (4.33–13.17%) [57], and for agricultural soils in the southwestern region of Ethiopia (5.81–6.85%) [15]. Total nitrogen (TN) varied across the sampling sites, indicating differences in soil fertility influenced by organic matter inputs, decomposition processes, fertilizer use, and land management practices, suggesting the need for targeted nitrogen management to support crop production. The available phosphorus values ranged from 10.28 to 27.21 mg/kg. A one-way ANOVA revealed a statistically significant difference in available phosphorus among the five sites (*p* < 0.05). Post hoc analysis using Tukey’s HSD test indicated that all pairwise comparisons between sites were significant (*p* < 0.05). The available phosphorus content in this study is lower than the range reported for Punjab, India (33–84 mg/kg [57], but higher than the range reported for soils in Awing, North West Cameroon (1.55–14.26 mg/kg) [53]. The substantially higher available phosphorus at B2 suggests potential differences in fertilizer application history, crop management practices, or nutrient input levels.

### 3.2. Potentially Toxic Elements (PTEs) Concentrations in Soils

**Figure 3:**
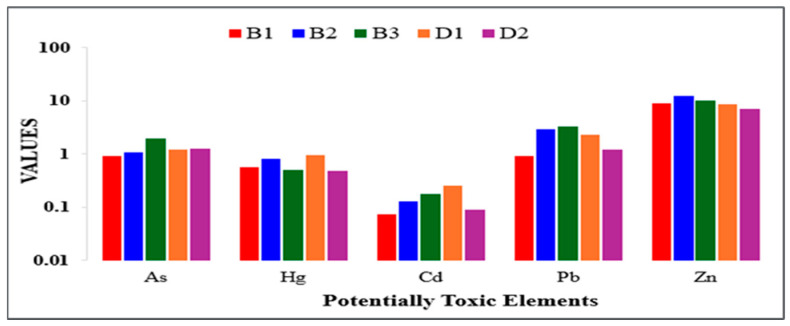
Comparison of PTEs concentrations in soil samples from five sampling sites (The value represents the average).

The concentrations of potentially toxic elements (PTEs) in agricultural soils of the North Gondar Zone varied across sampling sites (B1–D2), as shown in Table 2 and Figure 3. The metals were most abundant in the following order: Zn > Pb > Cd > As > Hg. Compared with the average concentrations reported for global soils [39], the studied agricultural soils samples exhibited higher Hg concentrations, whereas Pb, Cd, Zn, and As concentrations were relatively lower.

As concentrations ranged from 0.913 to 1.99 mg/kg, with the highest value observed at site B3. A one-way ANOVA revealed a statistically significant difference in As levels among the five sites (*p* < 0.05). These levels are well below the World Health Organization (WHO) permissible limit of 20 mg/kg for agricultural soils, indicating the absence of arsenic contamination [58]. Post hoc analysis using Tukey’s HSD test indicated that all pairwise comparisons between sites were significant (*p* < 0.05), except for the comparison between D2 and D1, which showed no significant difference (*p* > 0.05). The mean As concentration in this study is comparable to values reported for soils (0.18–1.72 mg/kg) [17] and for agricultural soils in the southwestern region of Ethiopia (0.930–1.12 mg/kg) [15]. However, the As levels observed in this study are significantly lower than those reported As concentrations in Ghana (95.70–390.75 mg/kg) [59], in the Nanchang District of Chongqing, China (3.12–10.56 mg/kg) [60], and in southeast China (18.2–29.6 mg/kg) [5].

The Hg concentration ranged from 0.493 to 0.953 mg/kg, with the maximum recorded at site D1. A one-way ANOVA revealed a statistically significant difference in Hg levels among the five sites (*p* < 0.05). Post hoc analysis using Tukey’s HSD test indicated that all pairwise comparisons between sites were significant (*p* < 0.05), except for the comparison between D2 and B3, which showed no significant difference (*p* > 0.05). The Hg levels in this study are significantly lower than those reported in Ghana (0.65 to 15.20 mg/kg) [59], in agricultural soils in the southwestern region of Ethiopia (4.97 to 8.63 mg/kg) [15], and in Bangladesh (133–5016 mg/kg) [61]. The Zn concentration ranged from 7.153 to 12.57 mg/kg, with the highest value observed at site B2. A one-way ANOVA revealed a statistically significant difference in Zn levels among the five sites (*p* < 0.05). Post hoc analysis using Tukey’s HSD test indicated that all pairwise comparisons between sites were significant (*p* < 0.05), except for the comparison between D1 and B1, which showed no significant difference (*p* > 0.05). The Zn concentration in this study is higher than that reported in the Wadi al Shati districts of Libya (0.256–6.802 mg/kg) [50]. However, the Zn levels in this study are significantly lower than those reported for agricultural soils in the southwestern region of Ethiopia (40.9–72.2 mg/kg) [15], Bangladesh (75.3–859.9 mg/kg) [61], Turkey (26.4–110 mg/kg) [62] and India (77.4 to 128 mg/kg) [63]. The zinc (Zn) concentration in the agricultural soil samples was below the established permissible limits [64]. The FAO/WHO recommended guideline value for Zn in soil is 300 mg/kg [65]. Therefore, Zn concentrations are within typical background levels for agricultural soils, likely influenced by fertilizers and organic amendments. However, they remain below standard guideline values, indicating no immediate risk to plant growth and human health.

The Cd concentration ranged from 0.073 to 0.26 mg/kg, with the highest value recorded at site D1. A one-way ANOVA revealed a statistically significant difference in Cd levels among the five sites (*p* < 0.05). Post hoc analysis using Tukey’s HSD test indicated that all pairwise comparisons between sites were significant (*p* < 0.05), except for the comparison between D2 and B1, which showed no significant difference (*p* > 0.05). These levels are well below the World Health Organization (WHO) permissible limit of 3 mg/kg for agricultural soils, indicating that the soils are uncontaminated with respect to cadmium [66]. The mean Cd concentration in this study is comparable to the range reported for soil in Pahang state, Malaysia (0.04 to 0.29 mg/kg) [17]. However, the Cd levels in this study are significantly lower than those reported for Indonesia (104.17–113.24 mg/kg) [67] and for agricultural soils in the southwestern region of Ethiopia (9.92–13.3 mg/kg) [15]. According to the joint FAO/WHO Codex Committee on Contaminants, the maximum permissible level of cadmium in agricultural soil is 3 mg/kg [65]. The Pb concentrations ranged from 0.933 to 3.31 mg/kg, with relatively maximum values in sites B2 and B3. A one-way ANOVA revealed a statistically significant difference in Pb levels among the five sites (*p* < 0.05). Post hoc analysis using Tukey’s HSD test indicated that all pairwise comparisons between sites were significant (*p* < 0.05). The mean lead (Pb) concentrations in the study ranged from 0.933 to 3.31 mg/kg, which is comparable to the range reported for uncontaminated soils (1.38–7.22 mg/kg) [17]. However, the Pb levels in this study are significantly lower than those reported for (5.4 to 65.4 mg/kg) [68], India (12.2 to 45.8 mg/kg) [63] and for agricultural soils in the southwestern region of Ethiopia (25.0–59.4 mg/kg) [15]. According to the joint FAO/WHO expert committee, the maximum tolerable limit of Pb in agricultural soil is established as 100 mg/kg [65].

### 3.3. Pearson Correlation Analysis

Pearson correlation analysis was conducted to assess the relationships among soil physicochemical properties (EC, MC, OC, OM, P, pH, and TN) and heavy metals (As, Hg, Cd, Pb, and Zn) showed in Figure S1. The resulting correlation coefficients indicated several strong positive and negative associations, suggesting common sources and similar geochemical behavior among certain variables. Moisture content (MC) showed apartial to moderate correlations with total nitrogen (TN) (r = 0.97), indicating that higher soil moisture is associated with increased nitrogen retention, likely due to enhanced organic matter decomposition and improved nutrient preservation conditions. OC and OM showed an almost perfect positive correlation (r = 0.99), indicating that organic matter is the main source of soil organic carbon and both vary nearly identically. Among the PTEs, Zn exhibited apartial to moderate correlations with phosphorus (P) (r = 0.95), suggesting a common anthropogenic origin, probable linked to fertilizer application and agricultural activities. Similarly, Pb showed apartial to moderate correlations with Zn (r = 0.71) and a moderate positive correlation with arsenic (As) (r = 0.64), indicating that these metals may share similar transport pathways or contamination sources. Cd was strongly associated with moisture content (MC) (r = 0.88) and total nitrogen (TN) (r = 0.89), suggesting that the observed relationship may be controlled by soil physicochemical properties, particularly soil organic matter, which significantly influences cadmium behavior through adsorption and complexation, leading to the formation of organometallic complexes that regulate its mobility and bioavailability. Mercury (Hg) also showed a strong positive correlation with Cd (r = 0.68), indicating similar environmental behavior or a common source of contamination. Electrical conductivity (EC) exhibited a moderate positive correlation with arsenic (As) (r = 0.55) and a very strong negative correlation with mercury (Hg) (r = −0.93). Moreover, EC was negatively correlated with cadmium (Cd) (r = −0.54) and total nitrogen (TN) (r = −0.39). These correlations indicate statistical associations among the measured variables; however, they should not be interpreted as evidence that electrical conductivity directly controls Hg accumulation, which is generally influenced by factors such as soil organic matter, mineral composition, and other environmental conditions. Organic carbon and organic matter exhibited showed apartial to moderate negative correlations with several heavy metals. Specifically, OC was strongly negatively correlated with Pb (r = −0.839) and Zn (r = −0.87), while OM showed similarly strong negative correlations with Pb (r = −0.835) and Zn (r = −0.87). These relationships suggest that Pb and Zn concentrations decrease as soil organic matter increases, indicating that their distribution is likely influenced by mineral composition, adsorption processes.

### 3.4. Pollution Assessment Indices

This section calculated PTEs contamination in agricultural soils using the geo-accumulation index, contamination factor, and pollution load index. These tools assess contamination levels, potential sources, and overall soil quality shown in (Table 3).

#### 3.4.1. Geo-Accumulation Index

**Figure 4:**
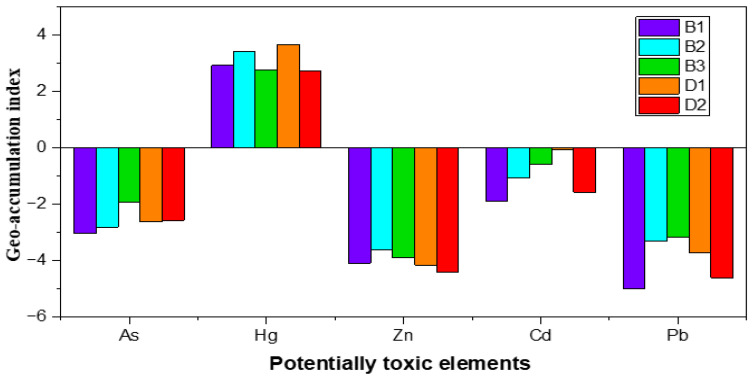
Geo-accumulation index (Igeo) showing the level of PTEs contamination in agricultural soils.

The Geoaccumulation Index (Igeo) was calculated to assess the degree of PTEs contamination in agricultural soil samples, as it quantifies contamination levels. Geo-accumulation index (Igeo) values varied between −5.00 and 3.67. All PTEs exhibited negative geoaccumulation index (Igeo) values, except for Hg, which showed positive values across all sampling sites. The Igeo values for the studied soils followed the order: Pb < Zn < As < Cd < Hg shown in Figure 4, indicating that Hg is the only element exhibiting significant anthropogenic accumulation, while all other metals remain within the unpolluted class. According to Müllers classification, agricultural soils at most sites were classified as unpolluted (Class 0; Igeo ≤ 0) for Zn, Cd, Pb, and As. In contrast, Hg fell within ranged from Class III (2 < Igeo ≤ 3; moderately to strongly polluted) to Class IV (3 < Igeo ≤ 4; strongly polluted) and also the contamination factor of Hg in all sampling sites CF ≥ 6 that is under class IV, very high contamination degree. A positive Igeo value indicates contamination, while a negative value indicates uncontaminated soil [69]. This elevated Hg can be attributed to agricultural inputs and natural sources, making it the dominant soil pollutant, whereas other potentially toxic elements remain at natural background levels.

#### 3.4.2. Contamination Factor

**Figure 5:**
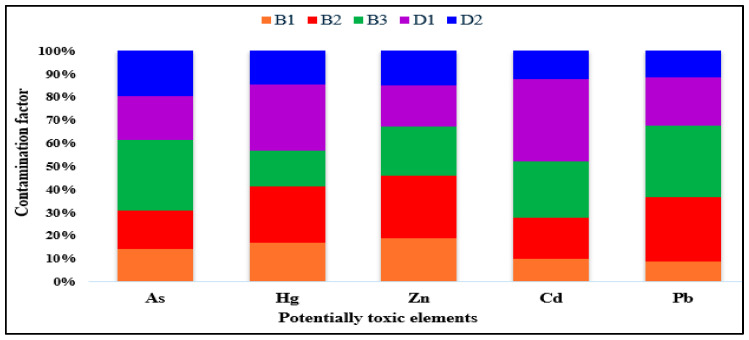
Contamination factor of PTE contamination in agricultural soils.

The CF index assesses PTEs contamination in agricultural soils. CF values ranged from 0.184 to 0.398 for As, 0.072 to 0.126 for Zn, 0.406 to 1.444 for Cd, 0.047 to 0.166 for Pb, and 9.86–19.06 for Hg. The contamination factor (CF) values for PTEs in soils followed the ascending order: Pb < Zn < Cd < As < Hg shown at (Figure 5). On the other hand, Hg levels significantly exceeded background values at all sites, indicating significant input from anthropogenic sources. Notably, the CFs of most other metals remained below 1, indicating minimal anthropogenic enrichment and low contamination levels. This classification of soils contaminated with PTEs is based on CF values: CF < 1 = low contamination factor; 1 ≤ CF < 3 = moderate contamination factor; 3 ≤ CF < 6 = considerable contamination factor; and CF ≥ 6 = very high contamination factor [70]. Higher CF values indicate a greater contribution from anthropogenic pollution sources, whereas lower values indicate predominantly natural origins, such as weathering of underlying bedrock [16].

#### 3.4.3. Pollution Load Index

**Figure 6:**
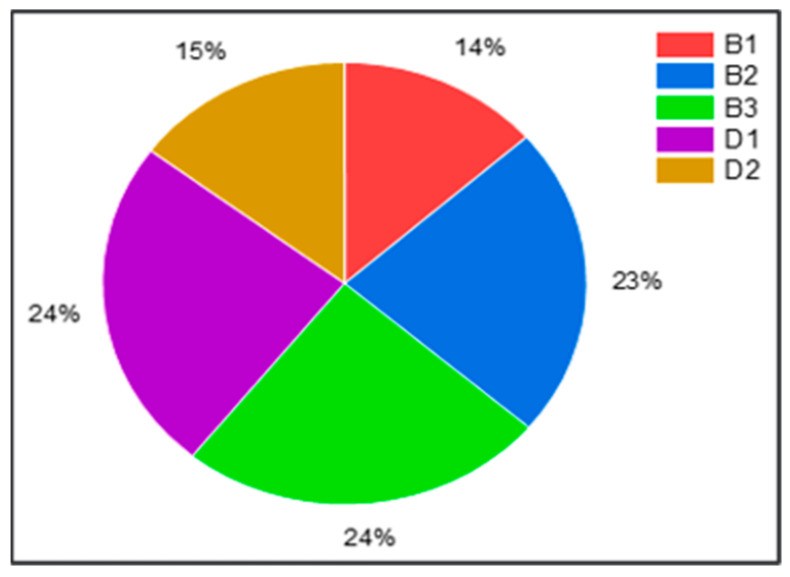
Pollution Load Index (PLI) across sampling site.

The Pollution Load Index (PLI) was used to assess the extent of PTEs in agricultural soils. The PLI values ranged from 0.32 to 0.58, with all sites showing values below the standard limits (PLI < 1), indicating unpolluted conditions and limited accumulation of PTEs in the agricultural soils. The spatial distribution of PLI (%) revealed heterogeneous contributions across the study area, with the highest shares at B3 and D1 (24.5% each), followed by B2 (22.8%), while the lowest values were observed at D2 (14.8%) and B1 (13.5%) shown in Figure 6. This spatial variability suggests differences in anthropogenic inputs, likely associated with variations in agricultural intensity and land management practices.

### 3.5. Health Risks Assessment

Potential health risks from PTEs in agricultural soil were assessed using chronic daily intake (CDI), hazard quotient (HQ), hazard index (HI), carcinogenic risk (CR), and total cancer Risk (TCR), as detailed in (Table 4, Table 5 and Table 6).

#### 3.5.1. Non-Carcinogenic Risk

The chronic daily intake (CDI) values of As, Hg, Cd, Zn, and Pb from Agricultural soil at sites B1, B2, B3, D1, and D2 for adults and children are shown in (Table 4).

The findings indicate that the estimated average chronic daily intake (CDI) of PTEs from agricultural soils followed the order Zn > Pb > As > Hg > Cd for both adults and children. For adults, the CDI values ranged from 4.52 × 10^−6^ to 7.93 × 10^−6^ mg kg^−1^ day^−1^ for Zn, 5.73 × 10^−7^ to 2.04 × 10^−6^ mg kg^−1^ day^−1^ for Pb, 5.81 × 10^−7^ to 1.27 × 10^−6^ mg kg^−1^ day^−1^ for As, 3.10 × 10^−7^ to 6.01 × 10^−7^ mg kg^−1^ day^−1^ for Hg, and 4.52 × 10^−8^ to 1.61 × 10^−7^ mg kg^−1^ day^−1^ for Cd. Among the investigated sites, B3 consistently exhibited the highest CDI values for As, Pb, and Zn, whereas D1 recorded the highest CDI for Hg and Cd, reflecting localized spatial variability in soil contamination. The CDI values for children ranged from 9.57 × 10^−5^ to 1.68 × 10^−4^ mg kg^−1^ day^−1^ for Zn, 1.25 × 10^−5^ to 4.43 × 10^−5^ mg kg^−1^ day^−1^ for Pb, 1.22 × 10^−5^ to 2.66 × 10^−5^ mg kg^−1^ day^−1^ for As, 6.59 × 10^−6^ to 1.28 × 10^−5^ mg kg^−1^ day^−1^ for Hg, and 9.76 × 10^−7^ to 3.48 × 10^−6^ mg kg^−1^ day^−1^ for Cd. The CDI values for all PTEs were consistently higher in children than in adults across all exposure pathways. This greater exposure is primarily attributable to children’s higher soil ingestion rates, lower body weight, and more frequent hand-to-mouth behavior, which increase their susceptibility to soil contaminants. The observed spatial variation in CDI further suggests localized anthropogenic influences, including the application of agrochemicals and atmospheric deposition, on the distribution of PTEs in agricultural soils. For all the determined PTEs, the estimated chronic daily intake (CDI) values were below the maximum tolerable daily intake (MTDI), indicating that the PTEs were at safe concentrations [71,72]. These findings highlight the necessity for targeted monitoring and preventive strategies for this vulnerable population.

**Figure 7:**
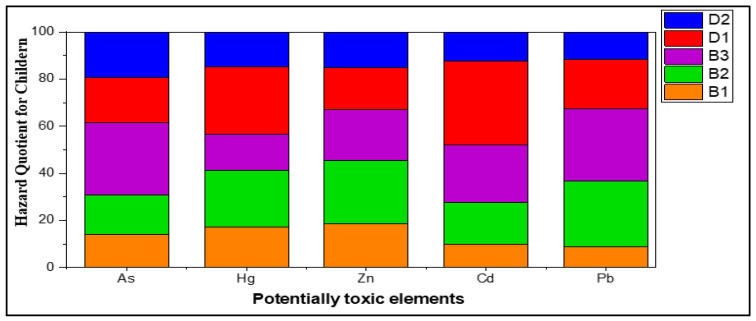
Hazard Quotient (HQ) for PTEs in Soil samples for Adults and Children.

The non-carcinogenic health risk associated with potentially toxic elements (PTEs) was evaluated using the hazard quotient (HQ) and hazard index (HI). The total HQ values for adults ranged from 2.0 × 10^−5^–3.0 × 10^−5^ for Zn, 2.2 × 10^−4^–7.9 × 10^−4^ for Cd, 6.7 × 10^−4^–1.30 × 10^−3^ for Hg, 2.1 × 10^−4^–7.3 × 10^−4^ for Pb, and 2.05 × 10^−3^–4.46 × 10^−3^ for As. For children, the corresponding HQ values ranged from 3.2 × 10^−4^–5.7 × 10^−4^ for Zn, 1.25 × 10^−3^–4.44 × 10^−3^ for Cd, 3.07 × 10^−3^–5.93 × 10^−3^ for Hg, 3.62 × 10^−3^–1.285 × 10^−2^ for Pb, and 4.085 × 10^−2^–8.904 × 10^−2^ for As. The HQ values for PTEs in agricultural soils were ranked in the following decreasing order: As > Hg > Cd > Pb > Zn for adults and As > Pb > Hg > Cd > Zn for children shown in Figure 7. For adults, the HQ values for Cd, As, Hg, Pb, and Zn in this study were lower than those reported for the southwest region of Ethiopia [15]. Similarly, the HQ values for Pb and Zn were lower than those reported for the Kpone landfill site in Ghana, whereas the HQ values for As and Hg were higher [59]. For children, the HQ values of Pb and Zn in this study were lower than those reported for the Kpone landfill site in Ghana, whereas the HQ values of As and Hg were higher [59]. Hazard quotient (HQ) values for all PTEs in the present study remained well below the threshold of 1, indicating negligible non-carcinogenic health risks associated with agricultural soil exposure both adults and children. The HQ values for all PTEs were higher in children than in adults. These results indicate that children are more susceptible to adverse effects from PTEs accumulation in agricultural soils because of their unique physiological characteristics and heightened vulnerability to soil-borne contaminants. Arsenic (As) was the dominant contributor to the cumulative risk, underscoring the need for targeted monitoring and preventive strategies for the most sensitive population.

The cumulative non-carcinogenic risk, expressed as the hazard index (HI), ranged from 3.28 × 10^−3^–6.47 × 10^−3^ for adults and 4.97 × 10^−2^–1.086 × 10^−1^ for children, decreasing across sites: B3 > D1 > B2 > D2 > B1. Site B3 showed the highest HI values for both population groups, indicating relatively greater cumulative exposure to PTEs at this location. However, all HI values remained well below the acceptable threshold of 1.0, suggesting negligible non-carcinogenic health risks from long-term soil exposure through ingestion and dermal contact.

#### 3.5.2. Carcinogenic Risk

**Figure 8:**
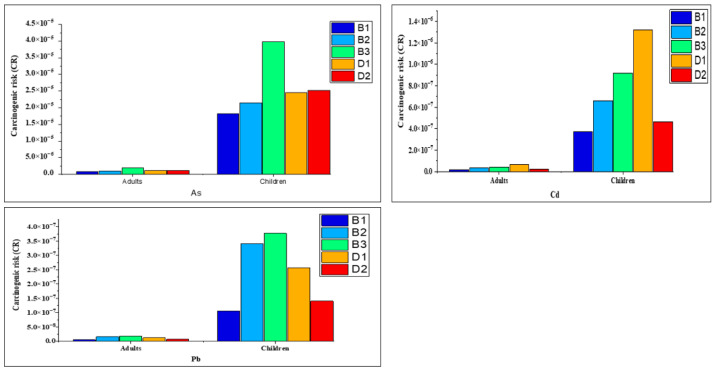
Carcinogenic risk (CR) of As, Cd, and Pb across sampling sites for adults and children.

Carcinogenic risks (CR) associated with PTEs exposure in agricultural soils were assessed by applying cancer risk shown in Figure 8 and total cancer risk (TCR) models separately for both adults and children.

The calculated carcinogenic risk (CR) values for adults ranged from 1.07 × 10^−6^–1.97 × 10^−6^ for As, 1.77 × 10^−8^–6.31 × 10^−8^ for Cd, and 5.05 × 10^−9^–1.62 × 10^−8^ for Pb. For children, CR values were higher, ranging from 1.83 × 10^−5^–3.99 × 10^−5^ for As, 3.71 × 10^−7^–1.32 × 10^−6^ for Cd, and 1.06 × 10^−7^–3.77 × 10^−7^ for Pb (Figure 7). For adults, the TCR ranged from 1.07 × 10^−6^ to 1.97 × 10^−6^, with site-specific CR contributions following the order As > Cd > Pb across all locations. For children, the TCR ranged from 1.87 × 10^−5^ to 4.04 × 10^−5^, which falls within the acceptable risk range of 1× 10^−6^ to 1 × 10^−4^ [45].The carcinogenic risk (CR) and total carcinogenic risk (TCR) for both adults and children remained within acceptable limits, indicating low to moderate cancer risk. In all cases, CR values for PTEs were higher in children than in adults. The TCR values in children were also higher than in adults, mainly due to greater exposure, higher soil ingestion rates, and lower body weight; however, all values remained within the acceptable range (10^−6^–10^−4^) according to the United States Environmental Protection Agency (USEPA) [73,74], indicating no significant carcinogenic risk. The overall carcinogenic risk followed the order B3 > D1 > D2 > B2 > B1, with B3 showing the highest risk and B1 the lowest. These results highlight that, although children are more vulnerable than adults, the overall cancer risk from exposure to PTEs in the studied agricultural soils is minimal.

## 4. Conclusions

This study presents an integrated assessment of PTEs in agricultural soils of the North Gondar Zone, Ethiopia, based on physicochemical properties, pollution indices, and human health risk evaluation. Physicochemical parameters (pH, electrical conductivity, organic carbon, organic matter, moisture content, total nitrogen, and available phosphorus) showed spatial variability. These moderately acidic to near-neutral pH conditions generally favor the availability of essential nutrients by maintaining balanced soil chemical conditions and supporting moderate rates of mineral weathering. The observed variation in soil pH may be attributed to differences in land use, vegetation cover, organic matter content, and parent material across the study sites. Electrical conductivity remained constant, indicating a non-saline environment suitable for agriculture. The statistical analysis revealed significant differences (*p* < 0.05) in all physicochemical parameters, except electrical conductivity, in sampling sites. The mean concentrations of PTEs varied significantly across the sampling sites, with the overall trend of Zn > Pb > Cd > As > Hg. Pollution assessment using the geoaccumulation index (Igeo), contamination factor (CF), and pollution load index (PLI), indicated that Zn, Cd, Pb, and As were within background levels (Igeo ≤ 0; CF < 1), whereas the geoaccumulation index (Igeo) indicated that Hg ranged from Class III (2 < Igeo ≤ 3; moderately to strongly polluted) to Class IV (3 < Igeo ≤ 4; strongly polluted) and also the contamination factor of Hg in all sampling sites CF ≥ 6 that is under class IV, very high contamination degree. Although the Pollution Load Index suggests overall unpolluted conditions. Correlation analysis reveals that PTE distribution is influenced by interactions among soil physicochemical properties, nutrient dynamics, anthropogenic inputs, and geochemical processes. The strong positive correlations among MC–TN (r = 0.973), P–Zn (r = 0.945), MC–Cd (r = 0.887), TN–Cd (r = 0.887), and Pb–Zn (r = 0.711) indicate possible common anthropogenic inputs and similar geochemical behaviors. Conversely, the strong negative correlations of OC and OM with Pb and Zn indicate that soil organic matter may influence the partitioning and availability of these metals. Health risk assessment shows that non-carcinogenic risks (HQ and HI) are below acceptable limits for both adults and children. However, carcinogenic risk analysis identifies arsenic as the main contributor to cancer risk. TCR values in children were higher than those of adults due to greater exposure and lower body weight but remained within the acceptable range (10^−6^–10^−4^), indicating a low to moderate cancer risk compared to adults. The general findings highlight the need for continuous monitoring of soil physicochemical properties and PTEs to ensure sustainable soil quality in the study area. Particular attention should be given to soil pH management, with liming of strongly acidic soils (pH < 5.5) to improve nutrient availability and reduce metal mobility. Integrated soil fertility management, including balanced fertilizer application and organic matter amendment, is also recommended to maintain soil health and minimize the accumulation and mobility of PTEs, particularly Hg. These practices are essential for maintaining long-term soil quality and ensuring environmental safety in agricultural systems.

## Figures and Tables

**Table 1:** Soil physicochemical parameters across sampled sites (mean ± SD, n = 3).

Parameters	Sample Site				
	B1	B2	B3	D1	D2
pH	6.72 ± 0.199	5.39 ± 0.224	6.21 ± 0.250	5.88 ± 0.440	5.36 ± 0.119
EC (μS/cm)	0.17 ± 0.046	0.15 ± 0.031	0.20 ± 0.043	0.12 ± 0.014	0.18 ± 0.112
OC (%)	1.97 ± 0.606	1.31 ± 0.467	1.20 ± 0.446	2.09 ± 0.092	2.44 ± 0.237
OM (%)	3.39 ± 1.045	2.25 ± 0.805	2.06 ± 0.769	3.62 ± 0.158	4.28 ± 0.516
MC (%)	9.80 ± 4.319	13.60 ± 1.497	20.00 ± 9.230	22.80 ± 9.376	17.60 ± 4.219
TN (%)	0.37 ± 0.016	0.40 ± 0.032	0.43 ± 0.0245	0.46 ± 0.016	0.42 ± 0.036
Available P (mg/kg)	13.10 ± 0.443	27.22 ± 0.206	14.32 ± 0.435	12.34 ± 0.156	10.28 ± 0.159

Note: mean ± SD: mean ± standard deviation; EC: Electrical Conductivity; OC: Organic Carbon; OM: Organic Matter; MC: Moisture Content; TN: Total Nitrogen; Available P: Available Phosphorus.

**Table 2:** PTEs concentrations in soil samples across sampling sites (mean ± SD, n = 3).

PTEs	Sampling Sites				
	B1	B2	B3	D1	D2
As	0.913 ± 0.025	1.07 ± 0.025	1.99 ± 0.10	1.23 ± 0.011	1.26 ± 0.01
Hg	0.57 ± 0.010	0.81 ± 0.026	0.513 ± 0.021	0.953 ± 0.025	0.493 ± 0.015
Zn	8.903 ± 0.057	12.57 ± 0.098	10.14 ± 0.026	8.493 ± 0.566	7.153 ± 0.215
Cd	0.073 ± 0.011	0.13 ± 0.010	0.18 ± 0.010	0.26 ± 0.010	0.091 ± 0.003
Pb	0.933 ± 0.021	2.997 ± 0.085	3.31 ± 0.026	2.267 ± 0.015	1.24 ± 0.01

**Table 3:** Geoaccumulation Index (Igeo), Contamination Factor (CF), and Pollution Load Index (PLI) of PTEs in soil samples.

Sites	Geo-Accumulation Index					Contamination Factors					Pollution Load Index
	As	Hg	Zn	Cd	Pb	As	Hg	Zn	Cd	Pb	PL1
B1	−3.04	2.93	−4.08	−1.89	−4.99	0.184	11.40	0.089	0.406	0.047	0.32
B2	−2.81	3.43	−3.60	−1.06	−3.32	0.214	16.20	0.126	0.722	0.150	0.54
B3	−1.91	2.77	−3.89	−0.59	−3.18	0.398	10.26	0.100	1.000	0.166	0.58
D1	−2.61	3.67	−4.15	−0.05	−3.73	0.246	19.06	0.085	1.444	0.113	0.58
D2	−2.57	2.72	−4.39	−1.57	−4.60	0.252	9.86	0.07	0.506	0.062	0.35

**Table 4:** Estimated Chronic daily Intake (CDIing + CDIderm) (mg/kg/day) of PTEs in Agricultural Soils.

Sites		Chronic Daily Intake (CDI)				
		As	Hg	Cd	Zn	Pb
B1	Adults	5.81 × 10^−7^	3.59 × 10^−7^	4.52 × 10^−8^	5.62 × 10^−6^	5.73 × 10^−7^
	Children	1.24 × 10^−5^	7.62 × 10^−6^	9.76 × 10^−7^	1.19 × 10^−4^	1.24 × 10^−5^
B2	Adults	6.81 × 10^−7^	5.09 × 10^−7^	8.05 × 10^−8^	7.93 × 10^−6^	1.84 × 10^−6^
	Children	1.43 × 10^−5^	1.08 × 10^−5^	1.73 × 10^−6^	1.68 × 10^−4^	4.01 × 10^−5^
B3	Adults	1.27 × 10^−6^	3.24 × 10^−7^	1.12 × 10^−7^	6.41 × 10^−6^	2.04 × 10^−6^
	Children	2.66 × 10^−5^	6.86 × 10^−6^	2.41 × 10^−6^	1.36 × 10^−4^	4.43 × 10^−5^
D1	Adults	7.83 × 10^−7^	6.01× 10^−7^	1.61 × 10^−7^	5.37 × 10^−6^	1.39 × 10^−6^
	Children	1.64 × 10^−4^	1.28 × 10^−5^	3.48 × 10^−6^	1.14 × 10^−4^	3.03 × 10^−5^
D2	Adults	8.03 × 10^−7^	3.10 × 10^−7^	5.60 × 10^−8^	4.52 × 10^−6^	7.59 × 10^−7^
	Children	1.68 × 10^−5^	6.59 × 10^−6^	1.21 × 10^−6^	9.57 × 10^−5^	1.66 × 10^−5^

**Table 5:** Non-carcinogenic Risk (HQ and HI) of PTEs in Agricultural Soils for Adults and Children.

Sites		Hazard Quotient (HQ)					HI
		As	Hg	Cd	Zn	Pb	
B1	Adults	2.05 × 10^−3^	7.80 × 10^−4^	2.20 × 10^−4^	2.00 × 10^−5^	2.100 × 10^−4^	3.28 × 10^−3^
	Children	4.085 × 10^−2^	3.550 × 10^−3^	1.250 × 10^−3^	4.000 × 10^−4^	3.620 × 10^−3^	4.970 × 10^−2^
B2	Adults	2.40 × 10^−3^	1.110 × 10^−3^	4.00 × 10^−4^	3.00 × 10^−5^	6.60 × 10^−4^	4.60 × 10^−3^
	Children	4.787 × 10^−2^	5.040 × 10^−3^	2.220 × 10^−3^	5.700 × 10^−4^	1.163 × 10^−2^	6.730 × 10^−2^
B3	Adults	4.460 × 10^−3^	7.000 × 10^−4^	5.500 × 10^−4^	2.000 × 10^−5^	7.300 × 10^−4^	6.47 × 10^−3^
	Children	8.904 × 10^−2^	3.190 × 10^−3^	3.070 × 10^−3^	4.600 × 10^−4^	1.285 × 10^−2^	1.086 × 10^−1^
D1	Adults	2.760 × 10^−3^	3.00 × 10^−5^	7.900 × 10^−4^	2.001 × 10^−5^	5.000 × 10^−4^	5.380 × 10^−3^
	Children	5.506 × 10^−2^	5.930 × 10^−3^	4.440 × 10^−3^	3.800 × 10^−4^	8.790 × 10^−3^	7.460 × 10^−2^
D2	Adults	2.050 × 10^−3^	6.700 × 10^−4^	2.800 × 10^−4^	2.010 × 10^−5^	2.700 × 10^−4^	4.06 × 10^−3^
	Children	5.640 × 10^−2^	3.070 × 10^−3^	1.550 × 10^−3^	3.200 × 10^−4^	4.810 × 10^−3^	6.620 × 10^−2^

**Table 6:** Carcinogenic risk (CR) and total cancer risk (TCR) of As, Cd, and Pb in agricultural soils for adults and children.

Sites		Carcinogenic Risk (CR)			Total Cancer Risk (TCR)
		As	Cd	Pb	
B1	Adults	8.72 × 10^−7^	1.77 × 10^−8^	5.05 × 10^−9^	1.070 × 10^−6^
	Children	1.83 × 10^−5^	3.71 × 10^−7^	1.06 × 10^−7^	1.87 × 10^−5^
B2	Adults	1.02 × 10^−6^	3.15 × 10^−8^	1.62 × 10^−8^	1.07 × 10^−6^
	Children	2.15 × 10^−5^	6.61 × 10^−7^	3.40 × 10^−7^	2.25 × 10^−5^
B3	Adults	1.91 × 10^−6^	4.37 × 10^−8^	1.79 × 10^−8^	1.97 × 10^−6^
	Children	3.99 × 10^−5^	9.16 × 10^−7^	3.77 × 10^−7^	4.04 × 10^−5^
D1	Adults	1.17 × 10^−6^	6.31 × 10^−8^	1.22 × 10^−8^	1.25 × 10^−6^
	Children	2.46 × 10^−5^	1.32 × 10^−6^	2.57 × 10^−7^	2.60 × 10^−5^
D2	Adults	1.20 × 10^−6^	2.20 × 10^−8^	6.70 × 10^−9^	1.23 × 10^−6^
	Children	2.52 × 10^−5^	4.64 × 10^−7^	1.40 × 10^−7^	2.57 × 10^−5^

## Data Availability

The original contributions presented in this study are included in the article/Supplementary Material. Further inquiries can be directed to the corresponding author.

## Supplementary Materials

The following supporting information can be downloaded at: https://www.mdpi.com/article/10.3390/toxics14070613/s1, Figure S1: Pearson correlation heatmap showing the relationships among soil physicochemical properties and PTEs.; Table S1: Selected wavelengths (λ), Correlation coefficients (R^2^), limit of detection (LOD), and limit of quantification (LOQ) by ICP -OES. Table S2: Recovery and precision test results for the laboratory control of Agricultural soil samples. Table S3: Definition and reference of some parameters for health risk assessment of heavy metal in soils [40,41,43,69,70]. Table S4: The reference doses of mg/kg/day for potentially toxic elements (PTEs) [41,70].

## References

[1] Wan Y., Liu J., Zhuang Z., Wang Q., Li H. (2024). Heavy metals in agricultural soils: Sources, influencing factors, and remediation strategies. Toxics.

[2] Yihune E., Addisu S. (2024). Assessment of Physicochemical Properties and Heavy Metal Content of Floriculture Soil in Amhara Region of Northwest Ethiopia. Sci. World J..

[3] Abitew M., Kebebew K. (2017). Physico-chemical characterization of soils for fertilizer recommendations for some districts in Bench-Maji Zone, South West, Ethiopia. Open Access J. Agric. Res..

[4] Hoque M.M., Islam A., Islam A.R.M.T., Pal S.C., Mahammad S., Alam E. (2023). Assessment of soil heavy metal pollution and associated ecological risk of agriculture dominated mid-channel bars in a subtropical river basin. Sci. Rep..

[5] Huang Y., Chen Q., Deng M., Japenga J., Li T., Yang X., He Z. (2018). Heavy metal pollution and health risk assessment of agricultural soils in a typical peri-urban area in southeast China. J. Environ. Manag..

[6] Ullah I., Ditta A., Imtiaz M., Mehmood S., Rizwan M., Rizwan M.S., Jan A.U., Ahmad I. (2020). Assessment of health and ecological risks of heavy metal contamination: A case study of agricultural soils in Thall, Dir-Kohistan. Environ. Monit. Assess..

[7] Ren Y., Lin M., Liu Q., Zhang Z., Fei X., Xiao R., Lv X. (2021). Contamination assessment, health risk evaluation, and source identification of heavy metals in the soil-rice system of typical agricultural regions on the southeast coast of China. Environ. Sci. Pollut. Res..

[8] Violante A., Cozzolino V., Perelomov L., Caporale A.G., Pigna M. (2010). Mobility and bioavailability of heavy metals and metalloids in soil environments. J. Soil Sci. Plant Nutr..

[9] Gu Y.G., Li Q.S., Fang J.H., He B.Y., Fu H.B., Tong Z.J. (2014). Identification of heavy metal sources in the reclaimed farmland soils of the pearl river estuary in China using a multivariate geostatistical approach. Ecotoxicol. Environ. Saf..

[10] Abdelhafez A.A., Li J. (2015). Environmental monitoring of heavy metal status and human health risk assessment in the agricultural soils of the Jinxi River area, China. Hum. Ecol. Risk Assess. Int. J..

[11] Pandey N., Tiwari A. (2021). Human health risk assessment of heavy metals in different soils and sediments. Heavy Metals in the Environment.

[12] Healy M.G., Ryan P.C., Fenton O., Peyton D.P., Wall D., Morrison L. (2016). Bioaccumulation of metals in ryegrass (*Lolium perenne* L.) following the application of lime stabilised, thermally dried and anaerobically digested sewage sludge. Ecotoxicol. Environ. Saf..

[13] Gelaye Y., Musie S. (2023). Impacts of heavy metal pollution on ethiopian agriculture: A review on the safety and quality of vegetable crops. Adv. Agric..

[14] Tchounwou P.B., Yedjou C.G., Patlolla A.K., Sutton D.J. (2012). Heavy metal toxicity and the environment. Molecular, Clinical and Environmental Toxicology and Environmental Toxicology.

[15] Etana E., Hussein R., Huluka A. (2025). Evaluation of some physicochemical parameters and health risks associated with potentially toxic elements (PTEs) in agricultural soils from the southwest region of Ethiopia. J. Hazard. Mater. Adv..

[16] Tomczyk P., Wdowczyk A., Wiatkowska B., Szymańska-Pulikowska A. (2023). Assessment of heavy metal contamination of agricultural soils in Poland using contamination indicators. Ecol. Indic..

[17] Islam M.S., Hassan F.U., Toriman M.E., Ahmad R., Bashir M.A., Rehim A., Raza Q.-U.-A., Ta G.C., Halim S.B.A. (2025). Spatial assessment and ecological risk evaluation of soil heavy metal contamination using multivariate statistical techniques. Catena.

[18] Alengebawy A., Abdelkhalek S.T., Qureshi S.R., Wang M.-Q. (2021). Heavy metals and pesticides toxicity in agricultural soil and plants: Ecological risks and human health implications. Toxics.

[19] Doabi S.A., Karami M., Afyuni M., Yeganeh M. (2018). Pollution and health risk assessment of heavy metals in agricultural soil, atmospheric dust and major food crops in Kermanshah province, Iran. Ecotoxicol. Environ. Saf..

[20] Roberts T.L. (2014). Cadmium and phosphorous fertilizers: The issues and the science. Procedia Eng..

[21] Franco A., Schuhmacher M., Roca E., Domingo J.L. (2006). Application of cattle manure as fertilizer in pastureland: Estimating the incremental risk due to metal accumulation employing a multicompartment model. Environ. Int..

[22] Neglo K.A.W., Gebrekidan T., Lyu K. (2021). The role of agriculture and non-farm economy in addressing food insecurity in Ethiopia: A review. Sustainability.

[23] Bouteska A., Sharif T., Bhuiyan F., Abedin M.Z. (2024). Impacts of the changing climate on agricultural productivity and food security: Evidence from Ethiopia. J. Clean. Prod..

[24] Tefera M., Solomon B., Guadie A., Lakew W., Sewachen B., Shumye D. (2025). Evaluating the contamination of soil, water and vegetables with heavy metals along with the estimation of transfer factor and human health risk in Gondar city, Ethiopia. Food Saf. Risk.

[25] IUSS Working Group WRB (2022). World Reference Base for Soil Resources. International Soil Classification System for Naming Soils and Creating Legends for Soil Maps.

[26] Taju M. (2021). Diversity, structure and regeneration status of woody species in Juniperus dominated dry Afromontane forest of Beyeda district, northern highlands of Ethiopia. Proc. Int. Acad. Ecol. Environ. Sci..

[27] Abebe E. (2011). Ethnobotanical Study on Medicinal Plants Used by Local Communities in Debark Wereda, North Gondar Zone, Amhara Regional State, Ethiopia. Master’s Thesis.

[28] Sparks D.L., Page A.L., Helmke P.A., Loeppert R.H. (2020). Methods of Soil Analysis, Part 3: Chemical Methods.

[29] Helrich K. (1990). Official Methods of Analysis of the Association of Official Analytical Chemists (AOAC).

[30] Walkley A. (1947). A critical examination of a rapid method for determining organic carbon in soils—Effect of variations in digestion conditions and of inorganic soil constituents. Soil Sci..

[31] Bray R.H., Kurtz L.T. (1945). Determination of total, organic, and available forms of phosphorus in soils. Soil Sci..

[32] Jaskuła J., Sojka M., Fiedler M., Wróżyński R. (2021). Analysis of spatial variability of river bottom sediment pollution with heavy metals and assessment of potential ecological hazard for the Warta river, Poland. Minerals.

[33] Müller G. (1969). Index of Geoaccumulation in Sediments of the Rhine River. GeoJournal.

[34] Kowalska J.B., Mazurek R., Gąsiorek M., Zaleski T. (2018). Pollution indices as useful tools for the comprehensive evaluation of the degree of soil contamination—A review. Environ. Geochem. Health.

[35] Orellana E.P., Custodio M., Bastos M.C., Ascencion J.C. (2020). Heavy metals in agriculture soils from high andean zones and potential ecological risk assessment in Peru’s central Andes. J. Ecol. Eng..

[36] Aschale M., Sileshi Y., Kelly-Quinn M., Hailu D. (2017). Pollution assessment of toxic and potentially toxic elements in agricultural soils of the city Addis Ababa, Ethiopia. Bull. Environ. Contam. Toxicol..

[37] Addis W., Abebaw A. (2017). Determination of heavy metal concentration in soils used for cultivation of *Allium sativum* L. (garlic) in East Gojjam Zone, Amhara Region, Ethiopia. Cogent Chem..

[38] Turekian K.K., Wedepohl K.H. (1961). Distribution of the elements in some major units of the earth’s crust. Geol. Soc. Am. Bull..

[39] Kabata-Pendias A. (2011). Trace Elements in Soils and Plants.

[40] Mohammadi A.A., Zarei A., Marjan E., Mahmoud T., Mahmood Y., Zahra Y., Fatemeh S., Safoura J. (2020). Assessment of heavy metal pollution and human health risks assessment in soils around an industrial zone in Neyshabur, Iran. Biol. Trace Elem. Res..

[41] Li B., Deng J., Li Z., Chen J., Zhan F., He Y., He L., Li Y. (2022). Contamination and health risk assessment of heavy metals in soil and ditch sediments in long-term mine wastes area. Toxics.

[42] Tomlinson D.L., Wilson J.G., Harris C., Jeffrey D. (1980). Problems in the assessment of heavy-metal levels in estuaries and the formation of a pollution index. Helgoländer Meeresunters..

[43] Fagbenro A., Yinusa T., Ajekiigbe K., Oke A., Obiajunwa E. (2021). Assessment of heavy metal pollution in soil samples from a gold mining area in Osun State, Nigeria using proton-induced X-ray emission. Sci. Afr..

[44] Saleh H.N., Panahande M., Yousefi M., Asghari F.B., Oliveri Conti G., Talaee E., Mohammadi A.A. (2019). Carcinogenic and non-carcinogenic risk assessment of heavy metals in groundwater wells in Neyshabur Plain, Iran. Biol. Trace Elem. Res..

[45] USEPA (2010). Human Health Risk Assessment: Risk-Based Concentration Table.

[46] Liu Y., Ma R. (2020). Human health risk assessment of heavy metals in groundwater in the luan river catchment within the north China Plain. Geofluids.

[47] USEPA (2002). Supplemental Guidance for Developing Soil Screening Levels for Superfund Sites [R]. Solid Waste and Emergency Response.

[48] Akter S., Yn J., Mj K., Km M. (2023). Analysis of Heavy Metals and Other Elements in Soil Samples for its Physicochemical Parameters Using Energy Dispersive X-Ray Fluorescence (EDXRF) Techniques. Austin J. Environ. Toxicol..

[49] Oyeyiola G., Agbaje A. (2013). Physicochemical analysis of a soil near microbiology laboratory at The University of Ilorin, main campus. J. Nat. Sci. Res..

[50] Salem M.A., Bedade D.K., Al-Ethawi L., Al-Waleed S.M. (2020). Assessment of physiochemical properties and concentration of heavy metals in agricultural soils fertilized with chemical fertilizers. Heliyon.

[51] Leiva-Tafur D., Goñas M., Culqui L., Santa Cruz C., Rascón J., Oliva-Cruz M. (2022). Spatiotemporal distribution of physicochemical parameters and toxic elements in Lake Pomacochas, Amazonas, Peru. Front. Environ. Sci..

[52] Rahman M., Ali M., Rahman M.A., Chandra P. (2024). Analysis of physico–chemical Parameters of soils collected from Brahmaputra river of Dhubri district, Assam, India. Afr. J. Biomed. Res..

[53] Mofor N.A., Tamungang E.B.N., Mvondo-zé A.D., Kome G.K., Mbene K. (2017). Assessment of physico-chemical and heavy metals properties of some agricultural soils of Awing-North West Cameroon. Arch. Agric. Environ. Sci..

[54] Rani J., Chaudhary S., Agarwal T. (2018). Assessment of Ph and moisture content in agricultural soils of Faridabad, Haryana. JETIR.

[55] Rabie R.K., Matter M.K., Khamis A.-E.-M.A., Mostafa M.M. (1985). Effect of salinity and moisture content of soil on growth, nutrient uptake and yield of wheat plant. Soil Sci. Plant Nutr..

[56] Pudełko A., Chodak M. (2020). Estimation of total nitrogen and organic carbon contents in mine soils with NIR reflectance spectroscopy and various chemometric methods. Geoderma.

[57] Bhatti S.S., Kumar V., Singh N., Sambyal V., Singh J., Katnoria J.K., Nagpal A.K. (2016). Physico-chemical properties and heavy metal contents of soils and kharif crops of Punjab, India. Procedia Environ. Sci..

[58] Codex Alimentarius Commission (2009). Joint FAO/WHO Food Standards Programme Codex Alimentarius Commission Thirty Third Session Geneva, Switzerland, 5–9 July 2010.

[59] Obiri-Nyarko F., Duah A.A., Karikari A.Y., Agyekum W.A., Manu E., Tagoe R. (2021). Assessment of heavy metal contamination in soils at the Kpone landfill site, Ghana: Implication for ecological and health risk assessment. Chemosphere.

[60] Yao C., Yang Y., Li C., Shen Z., Li J., Mei N., Luo C., Wang Y., Zhang C., Wang D. (2024). Heavy metal pollution in agricultural soils from surrounding industries with low emissions: Assessing contamination levels and sources. Sci. Total Environ..

[61] Rahman S.H., Khanam D., Adyel T.M., Islam M.S., Ahsan M.A., Akbor M.A. (2012). Assessment of heavy metal contamination of agricultural soil around Dhaka Export Processing Zone (DEPZ), Bangladesh: Implication of seasonal variation and indices. Appl. Sci..

[62] Vural H., Akbana A., Meral A. (2021). The effect of heavy metal pollution on urban ecosystem and the evaluation of different land classifications; in Bingöl city/Turkey. Manag. Environ. Qual. Int. J..

[63] Krishna A., Dasaram B. (2021). Assessing Potentially Toxic Elements (PTEs) Distribution and Behavior in Soils around an Agro-based Industries (India): Ecological Risk, Environmental and Analytical Inferences. Soil Sediment Contam. Int. J..

[64] FAO/WHO Codex Alimentarius Commission Food Additives and Contaminants (2001). Joint FAO/WHO Food Standards Programme.

[65] Joint FAO/WHO Food Standards Programme, Codex Committee on Contaminants in Foods (2011). Report of the Fifth Session of the Codex Committee on Contaminants in Foods.

[66] World Health Organization (2006). Guidelines for the Safe Use of Wastewater, Excreta and Greywater.

[67] Budianta W. (2021). THe influence of mineralogical composition on the adsorption capacity of heavy metals solution by java natural clay, Indonesia. ASEAN Eng. J..

[68] Gomaa F., Amin A.E.E.A.Z., El-Desoky M.A., Roshdy N.M., Usman A.R. (2024). Assessment of Ecological and Health Risks of Potentially Toxic Elements in Soil and Plant Under Long-Term Sewage Wastewater Irrigation. Bull. Environ. Contam. Toxicol..

[69] Parvez M.S., Nawshin S., Sultana S., Hossain M.S., Rashid Khan M.H., Habib M.A., Nijhum Z.T., Khan R. (2023). Evaluation of heavy metal contamination in soil samples around Rampal, Bangladesh. ACS Omega.

[70] Hakanson L. (1980). An ecological risk index for aquatic pollution control. A sedimentological approach. Water Res..

[71] US Environmental Protection Agency (2008). Health Effects Support Document for Boron.

[72] Gebeyehu H.R., Bayissa L.D. (2020). Levels of heavy metals in soil and vegetables and associated health risks in Mojo area, Ethiopia. PLoS ONE.

[73] New York State Department of Health (NYSDOH) (2011). Health Consultation: Public Comment Draft—Hopewell Precision Area Contamination.

[74] Willis B., Mann J., Ford R., Charp P., Wilder L. (1994). Environmental Data Needed for Public Health Assessments: A Guidance Manual.

