# Supplementary material for: Integrated Assessment of Potentially Toxic Elements (PTEs) Pollution in Agricultural Soils of North Gondar Zone, Ethiopia: Physicochemical Parameters, Pollution Levels, and Associated Health Risks
Source: Toxics. 2026 Jul 13;14(7):613. doi: 10.3390/toxics14070613 (PMC13431333; doi:10.3390/toxics14070613)
Supplement: Supplementary file 1 [file toxics-14-00613-s001.zip › toxics-4398277-supplementary.pdf]

Supporting Information Tables

Table S1. Selected wavelengths (λ), Correlation coefficients (R^2), limit of detection (LOD), and limit of quantification (LOQ) by ICP -OES

Metals	λ (nm)	R^2	LOD (mg/L)	LOQ (mg/L)
Fe	238.204	0.997	1.842	5.523
As	228.8	0.999	0.0004	0.0015
Hg	184.95	0.998	0.0079	0.0283
Zn	206.20	0.995	0.0064	0.0176
Pb	220.353	0.998	0.0323	0.1200
Cd	228.802	0.996	0.0026	0.0085

Table S2. Recovery and precision test results for the laboratory control of Agricultural soil samples

Metals	Conc. in sample before Spiked (mg/kg)	Added amounts (mg/kg)	Conc. in sample (mg/kg) after Spiked (mg/kg)	% Recovery	% RSD
Fe	379.61 ± 1.13	95	473.03± 6.09	98.33	7.07
As	1.29 ± 0.026	1	2.27± 0.036	108	1.80
Hg	0.69 ± 0.015	0.5	1.17 ± 0.015	96	5.35
Zn	10.01 ± 0.076	5	14.85 ± 0.13	96.77	3.67
Pb	2.14± 0.021	2.05	4.15 ± 0.025	97.72	1.49
Cd	0.16 ± 0.015	0.2	0.36 ± 0.02	98.34	2.89

Table S3. Definition and reference of some parameters for health risk assessment of heavy metal in soils [40,41,43,69 ,70]

Calculation formula	Definition	Unit	Value	
			Children	Adults
IngR	Ingestion rate	mg/day	200	100
SA	Skin Surface Area	cm^2/day	2800	5700
ED	Exposure duration	Year	6	30
AF	Skin adherence factor	mg/cm	0.2	0.07
BW	Body weight	kg	15	70
EF	Exposure frequency	days/year	365	365
ABS	Dermal absorption factor	unitless	0.001	0.01
AT	Average time	days	2190	25550

Table S4. The reference doses of mg/kg/day for potentially toxic elements (PTEs) [41, 70]

Elements	RfD_{ing}	RfD_{derm}
Zn	3×10^{-1}	6×10^{-2}
Pb	3.5×10^{-3}	5.25×10^{-4}
Cd	1×10^{-3}	1×10^{-5}
As	3×10^{-4}	1.23×10^{-4}
Hg	3×10^{-3}	2.1×10^{-5}

Supporting Information figure

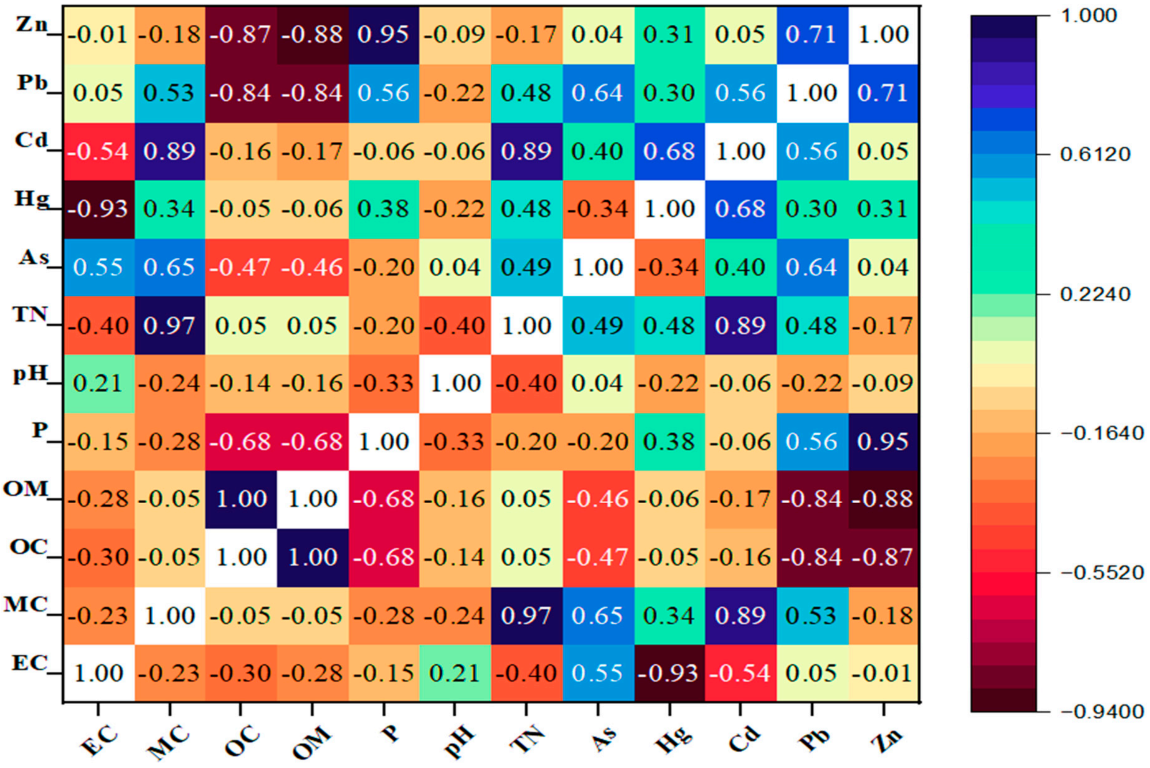

Figure S1 .Pearson correlation heatmap showing the relationships among soil physicochemical properties and PTEs.
